# TIM family gene polymorphism and susceptibility to rheumatoid arthritis: Systematic review and meta-analysis

**DOI:** 10.1371/journal.pone.0211146

**Published:** 2019-02-07

**Authors:** Bahman Razi, Samira Esmaeili Reykandeh, Shahab Alizadeh, AliAkbar Amirzargar, Amene Saghazadeh, Nima Rezaei

**Affiliations:** 1 Department of Hematology and Blood Banking, School of Allied Medical Sciences, Tehran University of Medical Sciences (TUMS), Tehran, Iran; 2 Molecular Immunology Research Center, Tehran University of Medical Sciences(TUMS), Tehran, Iran; 3 Department of Cellular and Molecular Nutrition, School of Nutritional Sciences and Dietetics, Tehran University of Medical Sciences (TUMS), Tehran, Iran; 4 Department of Immunology, School of Medicine, Tehran University of Medical Sciences (TUMS), Tehran, Iran; 5 Research Center for Immunodeficiencies, Children’s Medical Center, Tehran University of Medical Sciences, Tehran, Iran; 6 Systematic Review and Meta-analysis Expert Group (SRMEG), Universal Scientific Education and Research Network (USERN), Tehran, Iran; 7 Network of Immunity in Infection, Malignancy and Autoimmunity (NIIMA), Universal Scientific Education and Research Network (USERN), Tehran, Iran; University of Salamanca/University Hospital of Salamanca, SPAIN

## Abstract

**Background:**

TIM-family proteins are expressed on different immune cells such as dendritic cells, macrophages, type 1 and 2 T helper (Th) cells. Therefore, they have the ability to contribute to the various intracellular signals and immune responses, importantly the regulation of Th1 and Th17 cell differentiation, which plays a remarked role in fight against inflammatory and autoimmune diseases. Association of TIM family gene polymorphisms with rheumatoid arthritis (RA) has been frequently investigated. The findings however are not entirely consistent. Therefore, we carried out the present meta-analysis to examine the association between RA and the following TIM family gene polymorphisms: rs41297579, rs1036199, rs10515746, and rs7700944.

**Methods:**

A systematic search of Scopus, PubMed, and Web of Science databases was conducted through December 2018. Combined odds ratios (OR) with their corresponding 95% confidence intervals (CI) were calculated under different possible genetic models.

**Results:**

A total of eight case-control studies were included in the present meta-analysis. The results demonstrated significant association of RA with TIM-3 rs1036199 polymorphism under dominant (OR, 1.93, 95% CI, 1.43–2.61) and allelic models (OR, 1.74, 95% CI, 1.31–2.30). None of the other examined polymorphisms indicated significant association with RA.

**Conclusions:**

The present meta-analysis revealed that the TIM-3 rs1036199 polymorphism might confer susceptibility to RA. Further studies are required to reassert our findings.

## Introduction

Rheumatoid arthritis (RA) is an autoimmune disease characterized by progressive inflammation of the synovial membrane of the joint capsule and tendons (synovitis). Clinical manifestations range from chronic pain, loss of joint function, and deformity, to disability and systemic complications [1, 2]. RA usually develops between 40 and 50 years of age. Like most autoimmune diseases, RA is more common in women than men (3:1 ratio) [3]. Its prevalence increases with age and varies across different regions of the world [4, 5]. Despite clear clinical manifestations of the disease, the exact etiology and pathogenesis of RA remain obscure. Generally genetic and environmental factors have been well-associated with autoimmune disorders. Particularly, the role of genetic factors in the pathogenesis of RA has been confirmed by family and twin studies. Accordingly, the heritability of RA is estimated to be about 60% [6, 7]. Furthermore, genome-wide association studies (GWAS) have identified more than 100 genetic loci related to RA [8–12]. Of note, genome-side meta-analysis by Okada and colleagues recently introduced nine new loci including B3GNT2, ANXA3, CSF2, CD83, NFKBIE, ARID5B, PDE2A-ARAP1, PLD4 and PTPN2 as genetic risk factors for RA in Japanese population [12].

The transmembrane immunoglobulin and mucin domain (TIM) family gene was first isolated in 2001 [13]. This novel gene family has been described in both mice and humans. In mice, it is located on chromosome 11B1.1 and includes eight members (TIM-1–8). While in humans, it is located on chromosome 5q33.2 and includes three members: TIM-1, TIM-3, and TIM-4. [14]. Interestingly, the TIM gene family (5q33.2) is co-located with the interleukin-4 (IL-4) gene cluster that encodes cytokines including IL-3, IL-4, IL-5 and IL-13 on the human chromosome 5. This chromosomal co-location can partly explain shared function of TIM gene family and IL-4 gene cluster in the pathogenesis of autoimmune and allergic diseases.

TIM-family proteins belong to the type I transmembrane proteins consisting the N-terminal immunoglobulin (Ig) domain of the variable type, a mucin-like domain, and a C-terminal cytoplasmic tail [15, 16]. These proteins are expressed on different immune cells and therefore have the ability to mediate various intracellular signals. TIM-4 is found on macrophages and dendritic cells (DCs) [17, 18] while TIM-1 and TIM-3 are respectively expressed by T helper 2 (Th2) cells and T helper1 cells (Th1). It is, therefore, understandable that variants of the TIM gene family might interfere with signaling pathways related to Th1 and Th2 cells whereby the delicate balance between these cells is disturbed. This deviated balance has been implicates in a variety of allergic diseases and asthma [19, 20]. To date, TIM family gene polymorphisms have been associated with allergic rhinitis [21, 22], atopy [23, 24], asthma [22, 25–27], eczema [24], and autoimmune diseases such as multiple sclerosis [28, 29].

Several studies have investigated the association between RA and TIM family gene polymorphisms. The results are, however, not conclusive. To our knowledge, no meta-analysis has evaluated the association between RA and TIM gene polymorphisms. Therefore the present meta-analysis was conducted to address the issue whether the TIM family gene polymorphisms affect susceptibility to RA.

## Materials and methods

The present meta-analysis was performed in accordance with the Preferred Reporting Items for Systematic reviews and Meta-Analyses (PRISMA) statement (S1 File) and meta-analysis on genetic association studies checklist (S2 File) [30].

### Searches and data sources

We searched the Scopus, PubMed, and Web of Science databases through December 2018 by combining the following search terms: (“Arthritis, Rheumatoid” OR “Rheumatoid arthritis” OR RA) AND (“Hepatitis A virus cellular receptor-1” OR HAVCR-1 OR “T-cell immunoglobulin and mucin domain containing -1” OR TIM-1 OR “Kidney Injury Molecule -1” OR KIM-1 OR “Hepatitis A virus cellular receptor-2” OR HAVCR-2 OR “T-cell immunoglobulin and mucin domain containing -3” OR TIM-3 OR “T-cell immunoglobulin and mucin domain containing-4” OR TIM-4) AND (polymorphism OR genotype OR SNP OR “single nucleotide polymorphisms”). Studies were considered eligible if they investigated the association between RA and the following TIM family gene polymorphisms: rs41297579, rs1036199, rs10515746, and rs7700944. Further, we performed hand-searching of bibliographies from relevant publications to identify potential studies that were not retrieved by the electronic search. The search strategy was not confined to English language articles and any time period.

### Study selection

After removing duplicate records, two authors independently screened search results based on title and/or abstract. Then the apparently relevant papers were selected for detailed review. Finally, the authors carefully considered inclusion and exclusion criteria to include or exclude studies. Any disagreement between reviewers were resolved by consensus.

### Inclusion and exclusion criteria

Eligible studies should satisfy the following inclusion criteria: a) case–control studies that investigated the association between RA and the aforementioned TIM family gene polymorphism; and b) adequate data including genotype/allele frequency in both case and control groups were provided to calculate the combined odds ratio (OR) and their corresponding 95% confidence intervals (CIs). Book chapters, review articles, duplicate reports, conference abstracts, studies lacking control group, letters to the editor, case reports, and animal studies were all excluded.

### Data extraction and quality assessment

Two authors independently extracted the following data from each eligible study: the first author’s name, journal’s name, year of publication, ethnicity, country of origin, gender, study design, age of participants, methodology of genotyping, number of genotyped cases and controls, and genotype/allele frequency for in cases and controls. Genotype data were extracted for each ethnic group of people separately if data were available. Any disagreement between two reviewers were discussed and resolved by consensus. To assess the methodological quality of included studies, the Newcastle-Ottawa Scale (NOS) was used [31]. Studies with scores 0–3, 4–6 or 7–9 were of low, moderate or high-quality, respectively.

### Statistical analysis

Deviation from Hardy-Weinberg equilibrium (HWE) was analyzed using the Chi-Square test in control groups [32]. For each of the examined polymorphism, pooled odds ratios (ORs) and 95% confidence intervals (CIs) were calculated under different possible genetic models: dominant model, recessive model, allelic model, homozygote comparison and heterozygote comparison. To investigate the heterogeneity across included studies, we applied Cochran’s Q test and the I2 statistics [33]. There was significant heterogeneity if I2 values exceeded 50% or the Q statistic had a *p* value less than 0.1. If heterogeneity was detected, the random-effects model (Der Simonian–Laird approach) was applied. Otherwise, the fixed-effects model (Mantel–Haenszel approach) was used. Publication bias was assessed using the degree of funnel plot asymmetry and also by Begg’s test (a *p* value less than 0.05 was considered statistically significant) [34, 35]. The data analyses were carried out using STATA (version 14.0; Stata Corporation, College Station, TX) and SPSS (version 23.0; SPSS, Inc. Chicago, IL).

## Results

### Study characteristics

**Fig 1:**
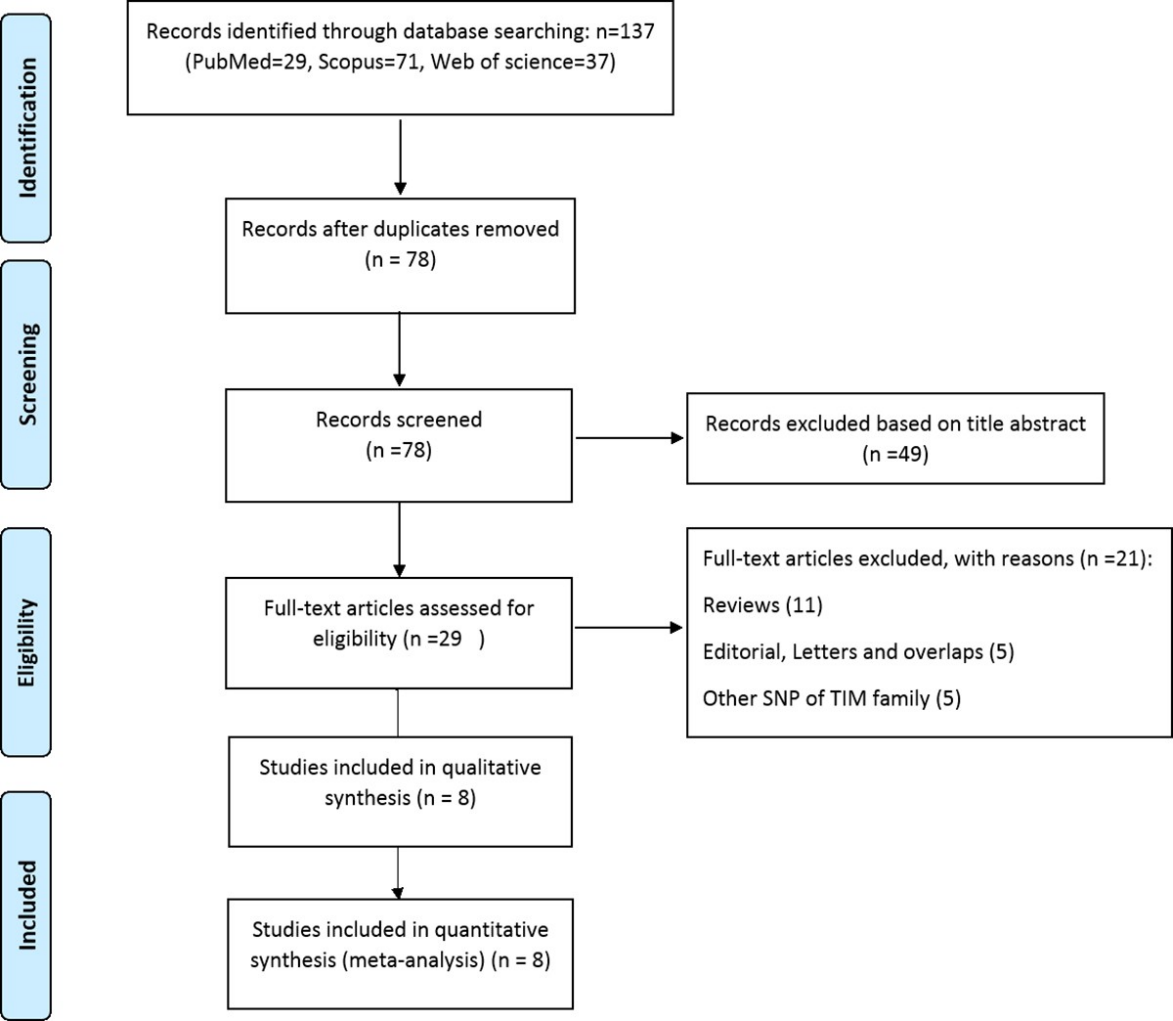
Flow diagram of study selection process.

Fig 1 displays search and screening process based on the PRISMA statement [36]. A total of 137 studies were examined. Eventually eight studies meeting inclusion and exclusion criteria were considered eligible to be included in the present meta-analysis. The studies were conducted in Korea [37–39], Egypt [40], China [41–43], and Iran [44]. All eligible studies had an overall good methodological quality with NOS scores ranging from 6 to 8. The RFLP (Restriction Fragment Length Polymorphism) was applied for genotyping SNPs in most studies. Tables 1 and 2 summarizes characteristics of the included studies.

**Table 1:** Characteristics of studies included in meta-analysis of overall RA.

Study author	Year	Country	Ethnicity	Sex cases/controls	Total cases/controls	Age case/control (Mean)	Genotyping method	Quality score
**TIM-1 rs41297579 G>A (-1454)**								
Chae et al.	2004	Korea	Asian	M = 43/203 F = 252/111	295/314	38,9/50,6	PCR–RFLP	7
Xu et al.	2012	China	Asian	M = NR F = NR	118/118	NR/NR	PCR–RFLP	6
Mosaad et al.	2015	Egypt	African	M = 25/NR F = 103/NR	128/125	46,9/49,1	PCR–RFLP	6
**TIM-3 rs1036199 G>T(+4259)**								
Chae et al.	2004	Korea	Asian	M = 43/194 F = 235/125	296/319	38,7/49,6	PCR	7
Song et al.	2011	Korea	Asian	M = 41/51 F = 325/338	366/389	51/47	RT-PCR	8
Xu et al.	2012	China	Asian	M = NR F = NR	226/231	NR/NR	PCR–RFLP	6
Xu et al.	2012	China	Asian	M = NR F = NR	103/108	NR/NR	PCR–RFLP	6
**TIM-3 rs10515746 T>G (-574)**								
Song et al.	2011	Korea	Asian	M = 41/51 F = 325/338	366/389	51/47	RT-PCR	8
Xu et al.	2012	China	Asian	M = NR F = NR	226/231	NR/NR	SSP-PCR	6
Xu et al.	2012	China	Asian	M = NR F = NR	103/108	NR/NR	SSP-PCR	6
**TIM-4 rs7700944**								
Xu et al.	2012	China	Asian	M = 117/102 F = 93/95	210/197	NR/NR	PCR–RFLP	7
Xu et al.	2012	China	Asian	M = 105/112 F = 109/99	214/211	NR/NR	PCR–RFLP	7
Zakeri et al.	2013	Iran	White	M = 16/35 F = 104/85	120/120	44,8/44,9	T-ARMS-PCR	6
Mosaad et al.	2015	Egypt	African	M = 25/NR F = 103/NR	128/125	46,9/49,1	PCR–RFLP	6

NR, not reported; M, male; F, female; RA, Rheumatoid Arthritis.

**Table 2:** Distribution of genotype and allele among RA patients and controls.

**Study author**	**RA cases**					**Healthy control**					**P-HWE**	**MAF**
	**GG**	**GA**	**AA**	**G**	**A**	**GG**	**GA**	**AA**	**G**	**A**		
**TIM-1 rs41297579 G>A (-1454)**					
Chae et al.	216	71	6	503	83	220	75	7	515	89	0.839	0.147
Xu et al.	86	26	4	201	35	90	26	2	205	31	0.938	0.127
Mosaad et al.	110	14	4	234	22	98	24	3	220	30	0.309	0.12
**Study author**	**RA cases**					**Healthy control**					**P-HWE**	**MAF**
	**TT**	**TG**	**GG**	**T**	**G**	**TT**	**TG**	**GG**	**T**	**G**		
**TIM-3 rs1036199 G>T(+4259)**					
Chae et al.	203	93	0	499	93	256	63	0	575	63	0/05	0/098
Song et al.	355	31	0	741	31	365	24	0	754	24	0/53	0/03
Xu et al.	198	28	0	424	21	244	7	0	455	7	0/822	0/013
Xu et al.	90	13	0	193	13	103	5	0	211	5	0/805	0/023
**Study author**	**RA cases**					**Healthy control**					**P-HWE**	**MAF**
	**GG**	**GT**	**TT**	**G**	**T**	**GG**	**GT**	**TT**	**G**	**T**		
**TIM-3 rs10515746T>G (-574)**					
Song et al.	343	23	0	709	23	378	11	0	767	11	0/777	0/014
Xu et al.	203	23	0	429	23	212	19	0	443	19	0/514	0/041
Xu et al.	100	3	0	203	3	85	21	2	191	25	0/602	0/115
**Study author**	**RA cases**					**Healthy control**					**P-HWE**	**MAF**
	**GG**	**GA**	**AA**	**G**	**A**	**GG**	**GA**	**AA**	**G**	**A**		
**TIM-4 rs7700944 G>A**					
Xu et al.	134	65	11	332	88	76	113	8	267	129	0	0/327
Xu et al.	77	135	2	289	139	135	70	6	341	85	0/387	0/194
Zakeri et al.	68	47	5	183	57	77	41	2	195	45	0/138	0/187
Mosaad et al.	29	87	12	145	111	99	23	3	221	29	0/25	0/116

P-HWE, p-value for Hardy–Weinberg equilibrium; MAF, minor allele frequency of control group

### Quantitative synthesis

#### TIM-1 rs41297579 polymorphism

**Fig 2:**
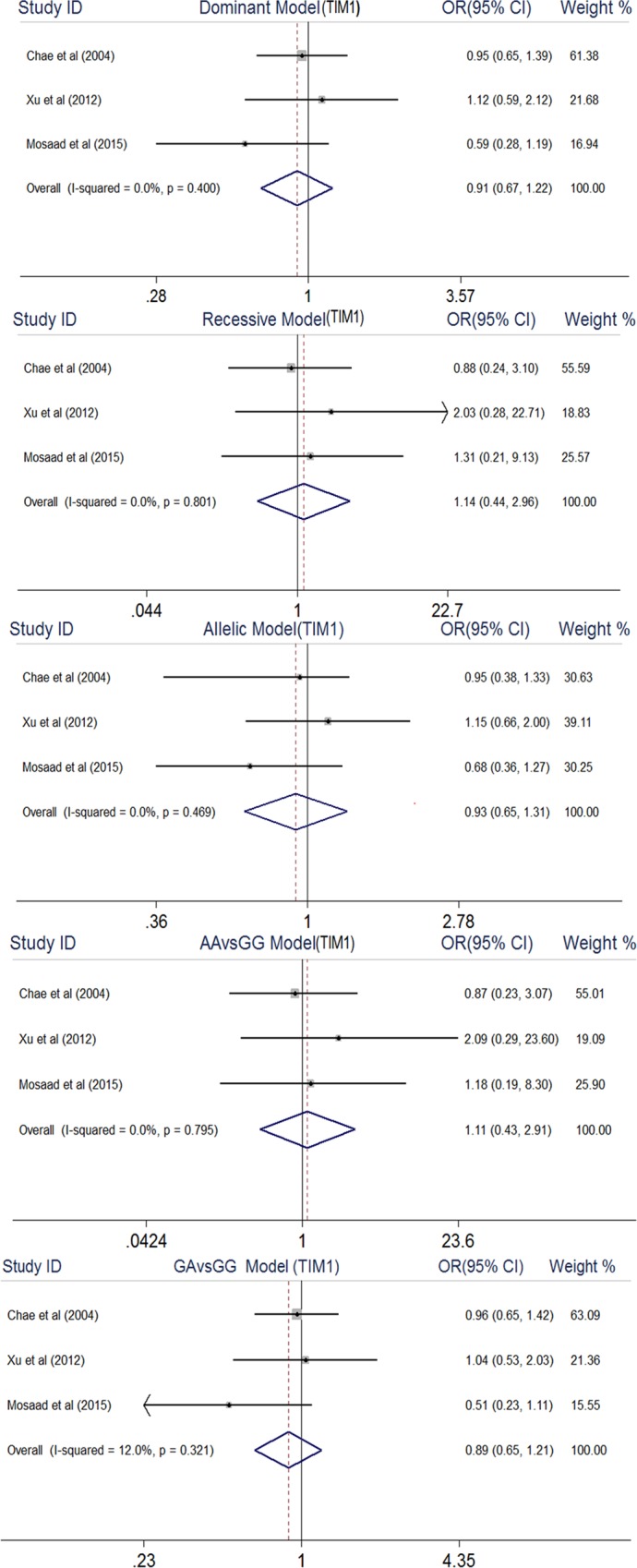
Forest plot of association between TIM1 rs41297579 gene polymorphism and rheumatoid arthritis.

As shown in Fig 2, meta-analysis found no significant association between TIM-1 rs41297579 polymorphism and RA under all genetic models: dominant model (OR, 0.91, 95% CI, 0.67–1.22), recessive model (OR, 1.14, 95% CI, 0.44–2.96), allelic model (OR, 0.93, 95% CI, 0.65–1.31), AA vs GG (OR, 1.11, 95% CI, 0.43–2.91), and GA vs GG (OR, 0.89, 95% CI, 0.65–1.21).

#### TIM-3 rs1036199 polymorphism

**Fig 3:**
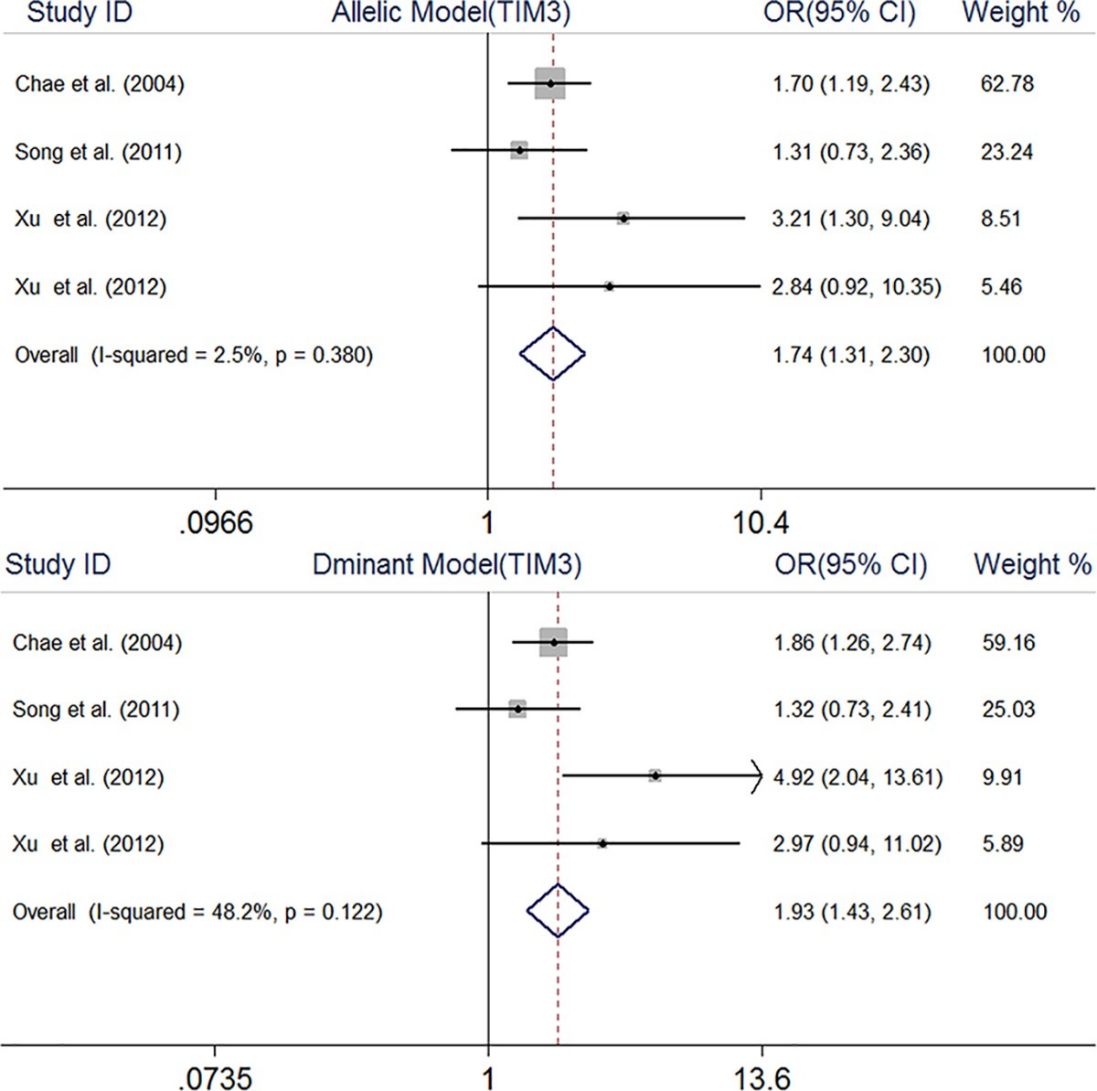
Forest plot of association between TIM3 rs1036199 gene polymorphism and rheumatoid arthritis.

The TIM-3 rs1036199 polymorphism was demonstrated to associate with RA risk under dominant model (OR, 1.93, 95% CI, 1.43–2.61) and allelic model (OR, 1.74, 95% CI, 1.31–2.30) (Fig 3). Due to the TIM-3 rs1036199 GG genotype frequency of zero in both cases and controls, the recessive model and GG vs TT model were not applicable and meta-analyses of comparisons related to the dominant (GG + TG vs TT) and heterozygote models (TG vs TT) resulted in the same findings. As Thakkinstian and colleagues described in [45], the dominant model was selected.

#### TIM-3 rs10515746 polymorphism

**Fig 4:**
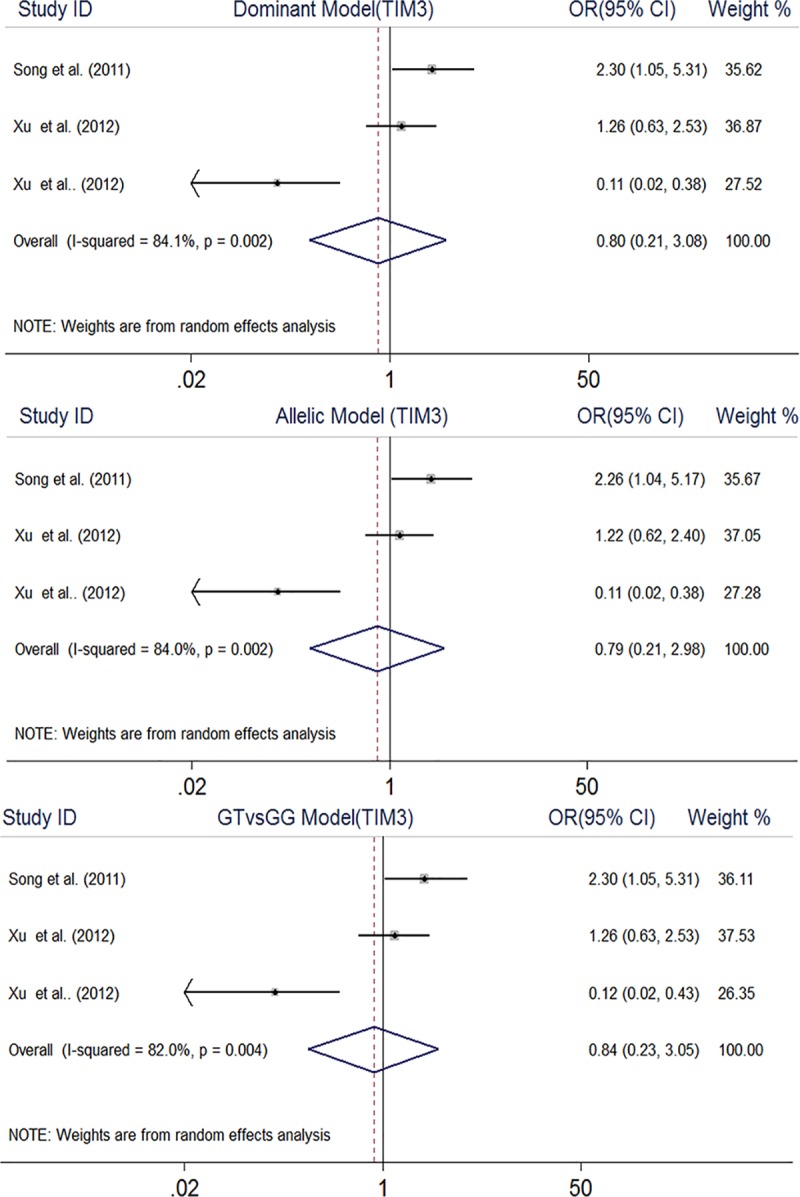
Forest plot of association between TIM3 rs10515746 gene polymorphism and rheumatoid arthritis.

The pooled results rejected any association between TIM-3 rs10515746 polymorphism and RA under dominant model (OR, 0.80, 95% CI, 0.21–3.08), allelic model (OR, 0.79, 95% CI, 0.21–2.98), and GT vs. GG model (OR, 0.84, 95% CI, 0.23–3.05) (Fig 4). Similar to the TIM-3 rs1036199 polymorphism, meta-analysis under other genetic models was not performed.

#### TIM-4 rs7700944 polymorphism

**Fig 5:**
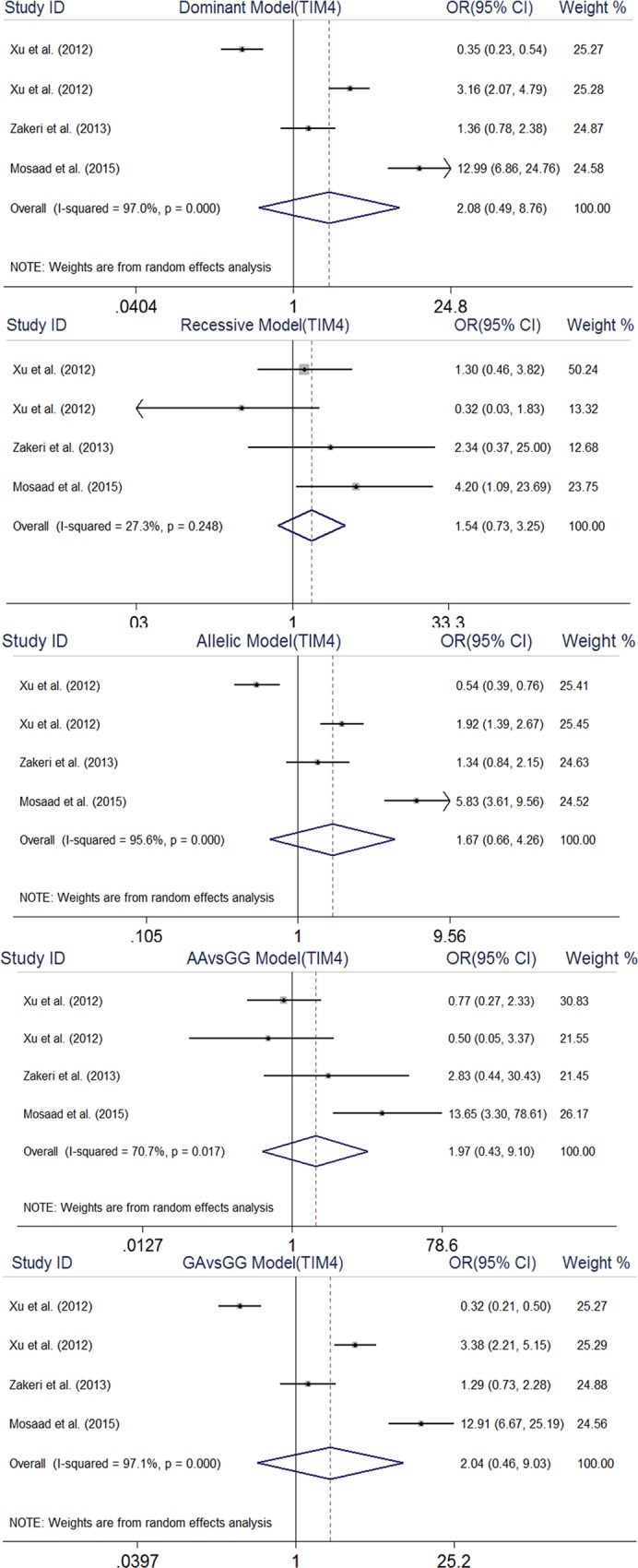
Forest plot of association between TIM4 rs7700944 gene polymorphism and rheumatoid arthritis.

No significant association was found between this polymorphism and RA: dominant model (OR, 2.08, 95% CI, 0.49–8.76), recessive model (OR, 1.54, 95% CI, 0.73–3.25), allelic model (OR, 1.67, 95% CI, 0.66–4.26), AA vs. GG model (OR, 1.94, 95% CI, 0.44–8.53), and GA vs. GG model (OR, 2.04, 95% CI, 0.46–9.03) (Fig 5).

### Evaluation of heterogeneity and publication bias

For the TIM-4 rs7700944 polymorphism, there was a significant heterogeneity across included studies under all genetic models, except for recessive model: dominant model (I2 = 97%), recessive model (I2 = 27.3%, *p* = 0.24), allelic model (I2 = 95.6%), AA vs GG model (I2 = 91.7%), and GA vs GG model (I2 = 97.1%). Also significant heterogeneity was observed across studies included in the three genetic models applicable for TIM-3 rs10515746: dominant model (I2 = 84.1%), allelic model (I2 = 84%), and GT vs GG model (I2 = 82%). No significant heterogeneity was detected among studies included in the meta-analyses related to other investigated SNPs. As shown in Table 3, the results of Begg’s test indicated no evidence of publication bias in the present meta-analysis. Also, funnel plots are presented in Supplementary Material (S1–S4 Figs).

**Table 3:** Main results of pooled ORs in meta-analysis of Tim family polymorphism.

	Sample size	Test of association		Test of heterogeneity		Test of publication bias (Begg’s test)	
Genetic model	Case/Control	OR	95% CI	I^2^ (%)	P	Z	P
**rs41297579**							
Dominant model	541/557	0.91	0.67–1.22	0	0.40	-0.52	0.602
Recessive model	541/557	1.14	0.44–2.96	0	0.80	1.57	0.117
Allelic model	541/557	0.93	0.65–1.31	0	0.46	-1.57	0.117
AA VS. GG	541/557	1.11	0.43–2.91	0	0.79	1.57	0.117
GA VS.GG	541/557	0.89	0.65–1.21	12	0.32	-1.57	0.117
**rs1036199**							
Dominant model	991/1047	1.93	1.43–2.61	48.2	0.12	0.68	0.497
Allelic model	991/1047	1.74	1.31–2.30	2.5	0.38	0.68	0.497
**rs10515746**							
Dominant model	695/728	0.80	0.21–3.08	84.1	0.002	-0.52	0.602
Allelic model	695/728	0.79	0.21–2.98	84	0.002	-0.52	0.602
GT VS.GG	695/728	0.84	0.23–3.05	82	0.004	-0.52	0.602
**rs7700944**							
Dominant model	672/653	2.08	0.49–8.76	97	≤0.001	0.68	0.497
Recessive model	672/653	1.54	0.73–3.25	27.3	0.24	0.0	1
Allelic model	672/653	1.67	0.66–4.26	95.6	≤0.001	0.68	0.497
AA VS. GG	672/653	1.94	0.44–8.53	91.7	≤0.001	0.68	0.497
GA VS.GG	672/653	2.04	0.46–9.03	97.1	≤0.001	0.68	0.497

## Discussion

Xie et al. 2017 [46] emphasized the association of TIM family gene polymorphisms, especially variants of the TIM1, with susceptibility to asthma through a meta-analysis study. Though some studies have investigated the association of RA with these polymorphisms, the associations remain unclear because the results are not conclusive. Therefore, we conducted this meta-analysis of data driven from eight studies that examined the relationship between RA and TIM family gene polymorphisms (rs41297579, rs1036199, rs10515746, rs7700944). Accordingly, the TIM-3 rs1036199 polymorphism was demonstrated to affect susceptibility to RA.

Analysis of the synovial fluid of RA patients revealed relatively high levels of pro-inflammatory cytokines such as IFN- γ, IL-6, IL-1, TNF-α, IL-23, and IL-12. These cytokines are able to attract leukocytes and activate joint cells such as osteoclasts and synovial fibroblasts. Activated joint cells stimulate the secretion of proteolytic enzymes, especially collagenase, which contribute to joint injury. Also, analysis of sera from patients with RA has often demonstrated the presence of rheumatoid factor (RF) or anti-citrullinated protein antibodies (ACPAs) [47]. It should however be noted that these autoantibodies are detected in various inflammatory diseases and their presence is, thus, not specific to RA. Moreover increased serum levels of C-reactive protein (CRP), an acute phase protein, are frequently found in patients with RA [48].

The TIM1−1454G>A polymorphism occurs in promoter region and therefore it is not expected to affect the function of TIM1 protein. Interestingly, Chae and colleagues reported that neither RF nor CRP were detectable in the sera of patients carrying this polymorphism [37].

The results showed significant association between TIM-3 G>T (+4259) and RA. The human TIM-3 gene coding region is consisted of seven exons. Among the various polymorphisms linked to this gene, the G>T (+4259) is the only SNP that occurs in the exon zone (exon 3). The change of allele at 4259 G>T leads to amino acid substitution from arginine to leucine. Arginine is an ionic amino acid whereas leucine is a non-polarized amino acid. Therefore the 4259 G>T polymorphism might alter the structure and function of gene. More interestingly, the TIM-3 protein is expressed on the surface of Th1 cells that serve to regulate Th1 and Th17 cell differentiation and their respective immune responses. Thus, the TIM-3 G>T (+4259) polymorphism might disturb the balance between Th1 and Th2 cell responses and thereby confer susceptibility to asthma and autoimmune diseases such as RA. Of note, Chae et al observed higher CRP values in the sera of patients harboring TIM1 +4259G>T variant [39].

The SNP rs7700944 is located in the intron region of the TIM-4 gene. The intron regions are comprised of noncoding DNA sequences which lie between coding regions in the genome. The intron regions play important role in the regulation and evolution of gene and control of alternative splicing in human genome. Evidences link polymorphisms that occur in the intron region of genes with different human diseases. For example, IL-1ß +3954 is an intronic polymorphism that has been associated with susceptibility to RA in a Chinese population [49]. After all, the present meta-analysis found no relationship between TIM-4 rs7700944 polymorphism and RA.

Although the present study was designed in a systematic manner to include all the eligible studies, there are some limitations to be mentioned. First is the concern about few number of included studies, because of which sensitivity and subgroup meta-analyses of studies stratified by sex, ethnicity, and methodology of genotyping could not be performed. Second that most of the included studies were conducted in East-Asians and so the findings are not generalizable to other race/ethnic groups. Third that our meta-analyses were confined to certain SNPs of TIM gene family due to inadequate numbers of publications.

## Conclusions

In sum, the current meta-analysis provided evidence that TIM-3 G>T (+4259) gene polymorphism might increase the risk of RA. The analyses, however, failed to found significant association between RA and other polymorphisms of TIM family genes. Further studies with larger sample sizes across different race/ethnic groups are warranted to validate the findings.

## Supporting information

S1 Fig Funnel plot for TIM1 rs41297579. (TIFF)

S2 Fig Funnel plot for TIM3 rs1036199. (TIFF)

S3 Fig Funnel plot for TIM3 rs10515746. (TIFF)

S4 Fig Funnel plot for TIM4 rs7700944. (TIFF)

S1 File PRISMA-DTA checklist. (DOC)

S2 File Meta-analysis on genetic association studies checklist. (DOCX)
