# Supplementary figures and images for: TIM family gene polymorphism and susceptibility to rheumatoid arthritis: Systematic review and meta-analysis
Source: PLoS One. 2019 Feb 7;14(2):e0211146. doi: 10.1371/journal.pone.0211146 (PMC6366744; doi:10.1371/journal.pone.0211146)

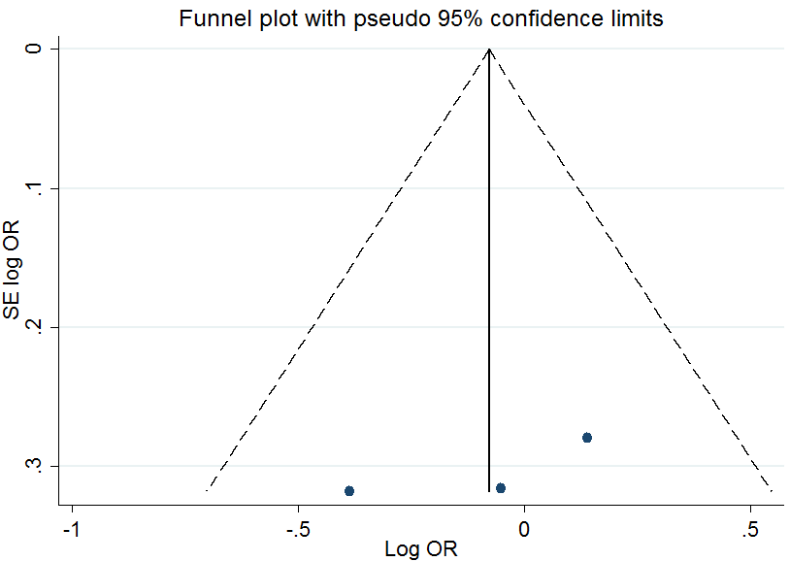

Supplement: S1 Fig — (TIFF) [file pone.0211146.s001.tiff]

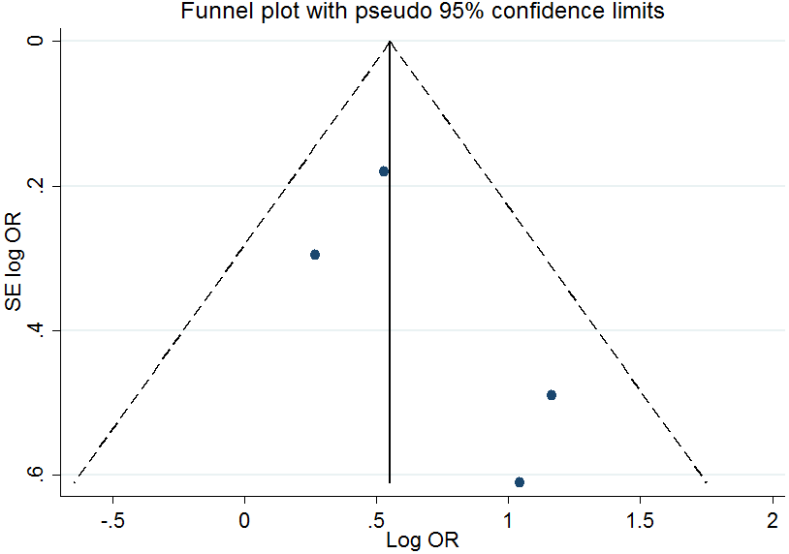

Supplement: S2 Fig — (TIFF) [file pone.0211146.s002.tiff]

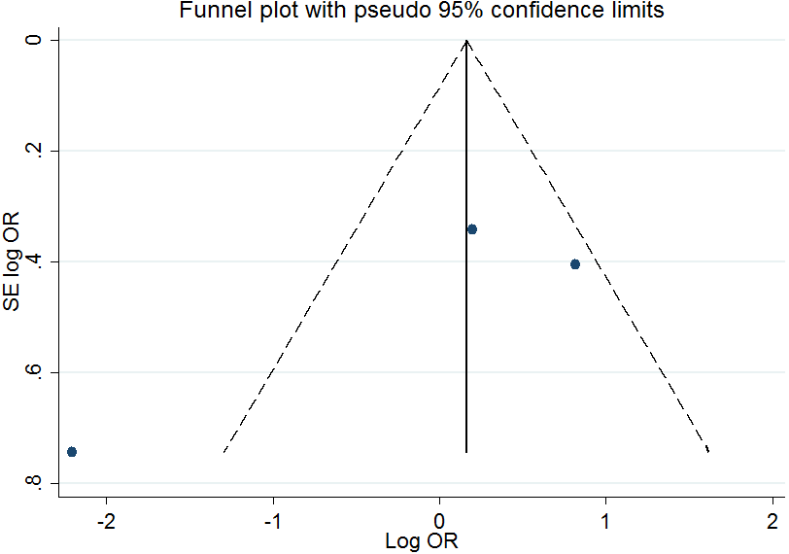

Supplement: S3 Fig — (TIFF) [file pone.0211146.s003.tiff]

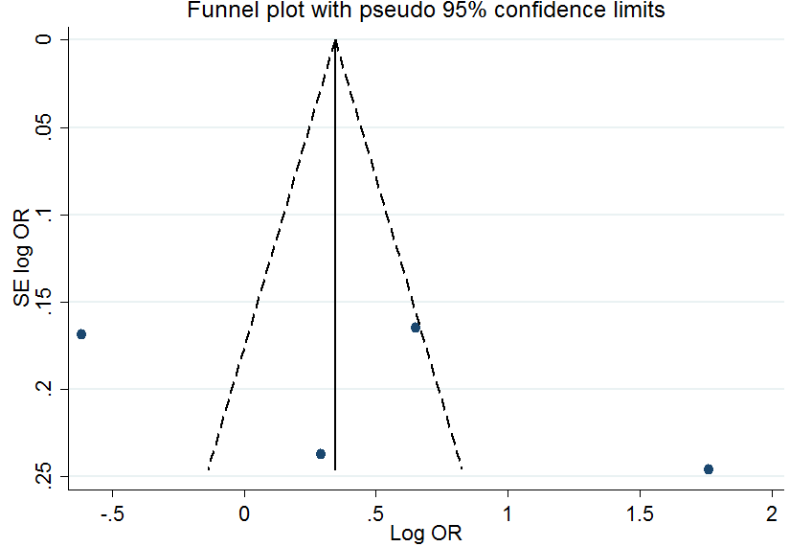

Supplement: S4 Fig — (TIFF) [file pone.0211146.s004.tiff]
